# Clinical application of the free anterolateral thigh chimeric muscle flap for reconstruction of composite tissue defects of the forearm and hand

**DOI:** 10.3389/fsurg.2026.1748013

**Published:** 2026-03-11

**Authors:** Shunan Dong, Jiyong Jiang, Rongyu Lan, Yuzhi Yu, Sijie Yang, Qikai Hua

**Affiliations:** 1 Department of Upper Extremity Trauma, Reconstruction and Plastic Surgery, Beijing Jishuitan Hospital Guizhou Hospital, Guiyang, China; 2 Department of Joint Surgery, The First Affiliated Hospital of Guangxi Medical University, Nanning, China; 3 Department of Hand Surgery, Guangzhou Heping Orthopedic Hospital, Guangzhou, Guangdong, China; 4 Department of Orthopedics, The People's Hospital of Guangxi Zhuang Autonomous Region, Nanning, China

**Keywords:** anterolateral thigh perforatorflap, chimeric flap, composite tissue defect, microsurgery, muscle flap

## Abstract

**Background:**

Composite tissue defects (CTD) of the forearm and hand commonly result from industrial, agricultural, and traffic-related injuries and often involve multiple anatomical structures, making reconstruction challenging. Simple skin-flap coverage may leave deep dead space and fail to restore functional integrity. This study reports our experience using the free anterolateral thigh chimeric muscle flap (ALT-CMF) for CTD reconstruction and evaluates its effectiveness in achieving reliable defect coverage and functional recovery.

**Methods:**

We retrospectively reviewed patients who underwent ALT-CMF reconstruction between February 2018 and January 2023. All patients had CTD of the forearm or hand and were followed for at least six months. Flap viability, postoperative complications, and donor-site outcomes were recorded. Digital functional recovery was assessed using the Total Active Motion (TAM) score. Two-point discrimination (2PD) were used to evaluate sensory recovery in the transplanted skin flaps.

**Results:**

Twenty patients (15 males, 5 females; mean age 39.40 ± 9.90 years) were included. During surgery, the median area of the harvested anterolateral thigh perforator skin flap was 80.0 (66.0–99.0) cm^2^, while the median area of the muscle flap component was 21.25 (20.00–30.00) cm^2^. All donor sites were primarily closed. Over a mean follow-up period of 15.30 ± 5.78 months, 15 flaps (75.0%) survived uneventfully, while 5 patients (25.0%) developed vascular crisis; four flaps were salvaged after urgent re-exploration, and one experienced complete necrosis. At the six-month follow-up, the mean TAM score was 235.79° ± 8.35°, indicating satisfactory recovery of digital mobility. 2PD testing demonstrated no significant difference in sensory recovery between the transplanted flaps and the contralateral side (19.42 ± 2.59 mm vs. 18.16 ± 3.20 mm, *P* = 0.063).

**Conclusions:**

The ALT-CMF is an effective reconstructive option for composite tissue defects of the forearm and hand. By providing reliable soft-tissue coverage, eliminating dead space through its chimeric muscle component, and supporting functional restoration in terms of joint mobility and sensation. This technique enables one-stage reconstruction with low donor-site morbidity and promotes favorable limb recovery.

## Introduction

Composite tissue defects (CTD) of the forearm and hand are common yet highly complex injuries, often resulting from high-energy trauma of various causes. These defects typically involve combined loss or damage of bone and multiple soft-tissue structures, making reconstruction particularly challenging. Without timely and appropriate management, patients may experience postoperative impairment in digital flexion and extension, severely compromising hand function. In more severe cases, functional disability or even amputation may occur, leading to loss of work capacity and substantial psychological burden for affected individuals (1–3).

Effective management of recalcitrant wounds accompanied by deep dead space hinges on the ability to adequately obliterate the cavity. When such injuries occur in the forearm and hand, reconstruction must not only address dead-space filling but also support subsequent restoration of hand function (4–6). Traditional approaches commonly involve debridement and negative-pressure wound therapy to promote granulation tissue formation, followed by skin grafting or flap coverage. However, these multistage strategies are time-consuming and fail to restore flexor–extensor function, often resulting in suboptimal outcomes (7–10).

Musculocutaneous flaps have also been used for these complex wounds, but the inseparable nature of the skin and muscle components limits flap mobility, making it difficult to simultaneously achieve reliable soft-tissue coverage, effective dead-space obliteration, and restoration of flexor–tendon continuity (2, 11). Surgeons have attempted the combined use of free skin flaps and free muscle flaps to treat wounds with deep cavities, but this technique requires harvesting and anastomosing two separate flaps, leading to prolonged operative time, greater technical complexity, increased donor-site and recipient-site morbidity, and substantially higher surgical risk (12, 13).

Advances in microsurgical perforator flap techniques have led to increasing reports of chimeric flap reconstruction for complex wounds in recent years. In 1991, Hallock first introduced the concept of the “chimeric flap,” referring to the harvest of multiple independent tissue components—each consisting of a single or composite tissue type—based on a single source vessel within the same vascular territory (14). This design minimizes donor-site morbidity while enabling precise three-dimensional reconstruction and functional restoration.

With the growing application of chimeric perforator flaps, they are now regarded as an optimal solution for three-dimensional reconstruction of composite tissue defects accompanied by deep dead space (15–17). For the recipient site, the relative independence of the skin and muscle components allows the muscle flap to be accurately inserted into the deep cavity and to bridge muscle defects, thereby contributing to functional restoration, while the skin flap provides reliable surface coverage. For the donor site, both components can be tailored in size and volume according to reconstructive needs, reducing donor-site morbidity Moreover, the entire reconstruction can be completed with a single set of vascular anastomoses, which may simplify the reconstructive strategy by avoiding multiple vascular anastomoses.

The anterolateral thigh flap (ALTF), first described in 1984, has since become one of the most widely used free flaps in microsurgical reconstruction. The free anterolateral thigh chimeric muscle flap (ALT-CMF) represents one of the earliest and most commonly applied chimeric flap variants (18–20). However, reports on the use of ALT-CMF for the reconstruction of CTD of the forearm and hand remain limited.

In this study, we retrospectively analyzed 20 patients treated at our institution between February 2018 and January 2023 who underwent single-stage reconstruction of forearm and hand CTD using the ALT-CMF. The findings provide new insight into the role of this chimeric flap in managing complex upper-extremity trauma.

## Methods

This retrospective study used clinical records and follow-up data retrieved from the Computer Information Center and the clinical follow-up system of Beijing Jishuitan Hospital Guizhou Hospital. All personal identifiers were removed to ensure participant confidentiality. The study protocol was approved by the **Ethics Committee of Beijing Jishuitan Hospital Guizhou Hospital.**

### Inclusion and exclusion criteria

Patients diagnosed with CTD of the forearm or hand who were treated at Beijing Jishuitan Hospital Guizhou Hospital between February 2018 and January 2023 were eligible for inclusion. All enrolled individuals were adults aged ≥18 years and underwent reconstruction using the ALT-CMF.

Patients were excluded if they met any of the following conditions: diagnosed with, or suspected of having, psychiatric disorders; history of diabetes mellitus; current treatment with glucocorticoids, immunosuppressive agents, or chemotherapy; presence of systemic infection; pre-existing arteriosclerosis or thromboangiitis obliterans; history of more than two prior flap procedures; ipsilateral limb amputation during follow-up; missing essential medical records; or a follow-up duration of less than six months.

These inclusion criteria were consistent with the clinical indications for ALT-CMF reconstruction in our institution. Accordingly, all patients who met these criteria and underwent ALT-CMF reconstruction during the study period were consecutively included in the analysis, thereby minimizing potential selection bias.

### Data collection and management

Baseline characteristics, treatment information, and follow-up data of all enrolled participants were collected. Demographic variables, including sex and age, as well as the mechanism and anatomical site of injury, were recorded at the time of admission.

During treatment, the dimensions of both the skin and muscle components of the flap were documented according to their actual harvested sizes. Follow-up records included wound-healing status, recovery of digital motion, and the occurrence of postoperative complications.

Postoperative digital mobility was assessed using Total Active Motion (TAM). Measurements were performed by a trained evaluator using a standard goniometer. Patients were instructed to perform maximal active finger flexion and extension. Active flexion angles of the metacarpophalangeal (MCP), proximal interphalangeal (PIP), and distal interphalangeal (DIP) joints were measured, and extension deficits at each joint were recorded. TAM was calculated according to the method recommended by the American Society for Surgery of the Hand (ASSH), defined as the sum of active flexion angles minus the sum of extension deficits across the MCP, PIP, and DIP joints (21). All measurements were performed by the same evaluator throughout the follow-up period to ensure consistency.

Postoperative sensory recovery of the transferred flap was evaluated using static two-point discrimination (2PD). Measurements were performed at 6 months postoperatively by a trained examiner in a quiet environment. A standardized two-point discriminator was applied perpendicular to the skin surface of the flap, with gradually decreasing distances between the two points. Patients were instructed to close their eyes and report whether they perceived one or two distinct points. The minimal distance at which two points could be consistently distinguished in at least three consecutive trials was recorded as the static 2PD value.

For comparison, static 2PD was also measured at the corresponding anatomical location on the contralateral, uninjured limb, which served as an internal control. In cases where reliable sensory testing could not be performed, the data were recorded as missing.

Patient-reported satisfaction was recorded as a supplementary subjective measure reflecting overall acceptance of treatment, rather than as a primary functional outcome.

### Surgical procedure

**Figure 1:**
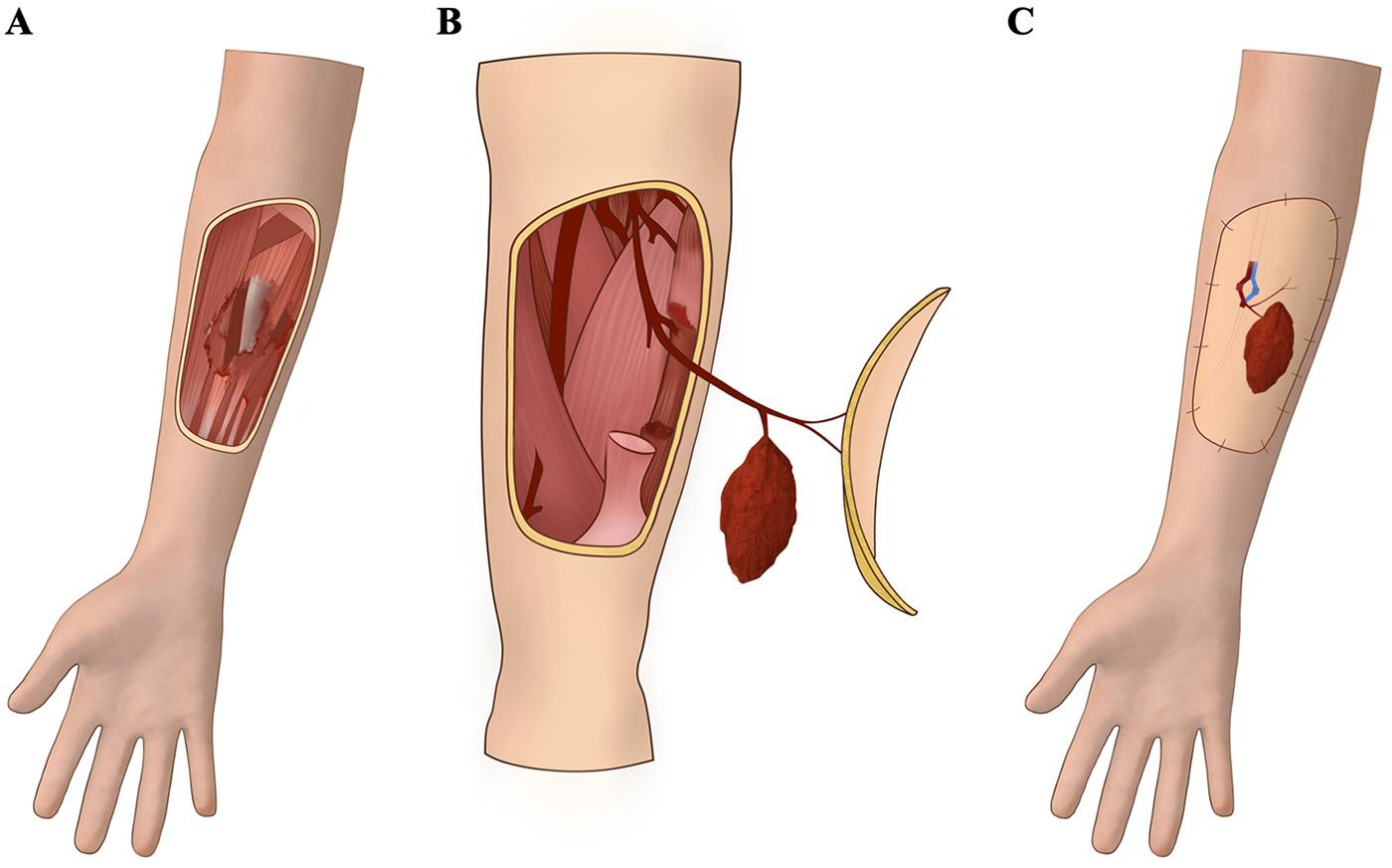
Schematic illustration of the operative procedure for the free anterolateral thigh chimeric muscle flap (ALT-CMF). **(A)** Thorough debridement of the injured area, followed by exposure and marking of the recipient arteries, veins, and the extent of muscle defect; **(B)** Harvesting of the ALT-CMF according to the requirements of the recipient site; **(C)** Transfer of the chimeric flap to the defect, with microsurgical anastomosis of the vascular pedicle to the recipient vessels and meticulous alignment and tension-adjusted suturing of the muscle component to the native muscle defect.

As showed in Figure 1, upon admission, all patients underwent emergency debridement, damage-control management, anti-infection therapy, and systemic supportive care. Intraoperatively, fractures were stabilized first, followed by thorough debridement of all necrotic tissue. The wound was repeatedly irrigated with copious saline. When complete debridement could not be achieved in a single stage, negative-pressure wound therapy was applied until the wound bed was clean and free of infection or necrosis, after which secondary reconstruction with the ALT-CMF was performed.

Preoperatively, all patients underwent computed tomographic angiography (CTA) of the lower limbs to identify perforators. The flap was designed along a line connecting the anterior superior iliac spine to the superolateral border of the patella. Using a handheld Doppler device, one to two cutaneous perforators arising from the descending branch of the lateral circumflex femoral artery were identified and marked as the centers for flap design. The skin paddle was tailored to exceed the wound dimensions by approximately 0.5 cm, with flap size adjusted as necessary to accommodate the depth and volume of the dead space.

All procedures were performed under general anesthesia. After induction, patients were positioned supine, and a tourniquet was applied to the upper arm of the affected limb. The recipient site was first prepared by routine debridement, including complete removal of necrotic tissue. The damaged muscles and tendons were identified, and the target recipient artery and vein were dissected. Under the operating microscope, adventitial trimming was performed to assess vessel quality.

Harvesting of the chimeric flap was then initiated following the preoperative design. Through a lateral approach, the skin paddle was elevated in the plane between the superficial and deep fat layers. When nerve reconstruction was required, the lateral femoral cutaneous nerve and its vascular supply were preserved within the flap. The fascia lata was incised along the perforator pathway, exposing the vastus lateralis muscle. Dissection was continued deeper to identify the descending branch of the lateral circumflex femoral artery (LCFA). From the medial aspect, sequential dissection was performed to connect with the lateral field, allowing complete skeletonization of the perforators. Depending on the size of the recipient-site dead space, a segment of the vastus lateralis muscle with an independent perforator was harvested to form the muscle component of the chimeric flap. After meticulous hemostasis, the donor site was closed primarily whenever possible. If primary closure was not feasible, a split-thickness skin graft harvested from the groin was applied and secured with compression dressing.

The harvested chimeric flap was transferred to the recipient site. The muscle flap was carefully inset into the deep muscular defect and sutured to the adjacent native muscle to maintain appropriate physiological tension and muscle length, thereby reducing the risk of postoperative atrophy. The muscle component was positioned to restore structural continuity rather than to function as an active contractile unit. Under microscopic visualization, the vascular pedicle of the skin paddle was anastomosed to the selected recipient artery and its accompanying vein. For arterial defects, flow-through anastomosis was performed. Patency of all anastomoses was confirmed by reperfusion testing. Finally, incisions were closed, and postoperative analgesia was provided as needed.

A closed-suction drain was placed beneath each flap and removed when the drainage decreased and became clear, typically within 3–6 days. The affected limb was immobilized in a functional position using a plaster cast.

### Postoperative management

Postoperatively, patients were advised to abstain from smoking, maintain adequate warmth, and remain on bed rest with the affected limb elevated to heart level. Standard postoperative care included antibiotic prophylaxis, anticoagulation therapy, vasospasm prevention, and analgesia. Flap perfusion was monitored closely, and dressings were changed regularly. Skin sutures were removed 12–14 days after surgery. Once flap viability was confirmed, patients were instructed to begin graduated functional rehabilitation.

### Statistical analysis

This retrospective study was analyzed using SPSS version 26.0 (IBM Corp., Armonk, NY, USA). Continuous variables were first assessed for normality using the Shapiro–Wilk test. Data with a normal distribution were presented as mean ± standard deviation (SD), whereas non-normally distributed data were expressed as median with interquartile range (IQR). Categorical variables were reported as counts and percentages [*n* (%)]. For paired data analyses, paired Student's t-tests or Wilcoxon signed-rank tests were applied as appropriate. A two-sided *P* value < 0.05 was considered statistically significant. All other analyses were descriptive in nature.

## Results

A total of 20 patients met the inclusion criteria for this study, including 15 males and 5 females, with a mean age of 39.40 ± 9.90 years. The injured limb was on the left side in 6 patients and on the right side in 14 patients. The mechanisms of injury included traffic accidents in 10 cases, machine-related injuries in 8 cases, and falls from height in 2 cases.

12 patients presented with fractures or defects of the ulna and/or radius; 8 patients had laceration or loss of the ulnar and/or radial artery; and 5 patients sustained injuries involving the ulnar and/or radial nerve. Intraoperatively, complete nerve discontinuity was confirmed in several patients with nerve injuries at the recipient site. All patients exhibited varying degrees of wrist and hand motor and sensory dysfunction. Detailed patient characteristics are summarized in Table 1.

**Table 1:** Basic characteristics of the 20 patients with composite tissue defects (CTD) of the forearm and hand.

Variable	Value	*P* value
Sex		
Male	15 (75.00)	
Female	5 (25.00)	
Total	20 (100)	
Age (years)	39.40 ± 9.90	
Injury mechanism		
Traffic accident	10 (50.00)	
Mechanical injury	8 (40.00)	
Fall from height	2 (10.00)	
Location		
Left	6 (30.00)	
Right	14 (70.00)	
Ffracture	12 (60.00)	
Vascular injury	8 (40.00)	
Nerve injury	5 (25.00)	
Flap size (cm^2^)	80.0（66.0, 99.0）	
Muscle flap size (cm^2^)	21.25 (20.00, 30.00)	
Complications		
Vascular crisis crisis	5 (25.00)	
flap necrosis	1 (5.00)	
Follow-up (months)	15.30 ± 5.78	
TAM at 6 months (°)^a^	235.79 ± 8.35	
2PD at 6 months (mm)^a^		
Flap side	19.42 ± 2.59	0.063^b^
Contralateral side	18.16 ± 3.20	

Data are presented as *n* (%), mean ± SD, or median (interquartile range), as appropriate.

^a^ TAM and 2PD were available for 19 of the 20 patients.

^b^ *P* value for 2PD was calculated using a paired Student's t-test.

During surgery, the mean area of the harvested anterolateral thigh perforator skin flap was 80.00 (66.00, 99.00) cm^2^, while the mean area of the muscle flap component was 21.25 (20.00, 30.00) cm^2^.

Because of differences in injury mechanisms and wound contamination, some patients required serial debridement and infection control before definitive ALT-CMF reconstruction, whereas others were suitable for immediate reconstruction. Consequently, the interval between injury and definitive reconstruction varied among patients.

After a mean follow-up period of 15.30 ± 5.78 months, all donor sites achieved primary closure. A total of 19 flaps (95.00%) survived, while 1 flap (5.00%) developed vascular crisis and progressed to necrosis despite urgent surgical re-exploration.

During follow-up, 5 patients (25.00%) developed vascular crisis of the flap. After urgent surgical exploration and salvage attempts, 4 flaps were successfully preserved. At 6 months postoperatively, TAM assessment—excluding the single case of flap necrosis—showed good digital functional recovery, with a mean TAM of 235.79° ± 8.35°. Sensory evaluation using static two-point discrimination (2PD) demonstrated acceptable sensory recovery of the transferred flaps, with a mean 2PD of 19.42 ± 2.59 mm on the flap side, which was comparable to that of the contralateral side (18.16 ± 3.20 mm), showing no statistically significant difference (*P* = 0.063). 19 patients (95.00%) reported satisfaction with the aesthetic appearance and functional recovery of the affected limb.

### Representative cases

We present 2 representative cases to illustrate the typical therapeutic course and clinical outcome associated with ALT-CMF.

#### Case 1

**Figure 2:**
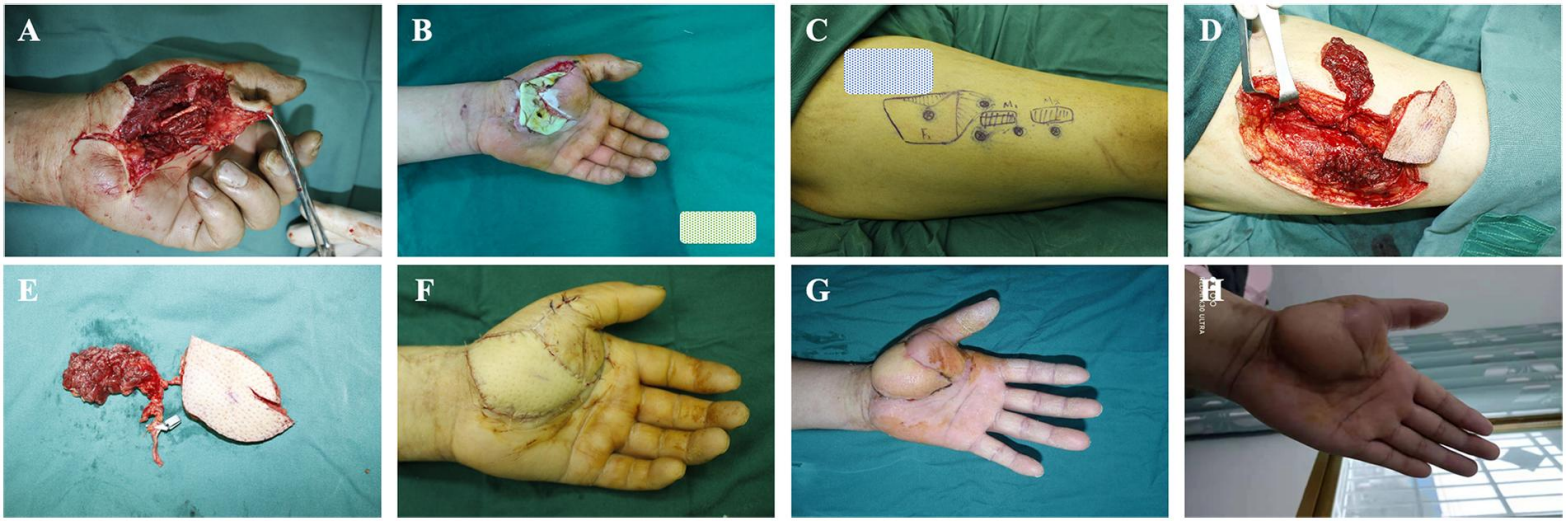
Application of the ALT-CMF in repairing a palmar composite tissue defect (CTD). **(A)** High-pressure injection injury resulting in a palmar thenar skin and soft-tissue defect; **(B)** Temporary coverage with antibiotic-loaded bone cement for infection control; **(C–E)** Preoperative design and intraoperative harvest of the ALT-CMF; **(F)** Immediate postoperative appearance of the reconstructed left palm; **(G)** Appearance of the flap one month after surgery; **(H)** Appearance at six months postoperatively.

A 40-year-old man sustained a high-pressure injection injury causing a skin and muscle defect with infection over the left thenar eminence (Figure 2A). Following thorough debridement, antibiotic-loaded bone cement was applied to temporarily cover the defect (Figure 2B). Two weeks later, a free right ALT-CMF was harvested and used to reconstruct the palmar defect (Figures 2C–F). At one month postoperatively, the flap showed complete survival (Figure 2G). By six months, the muscle defect had fully healed, and hand function had recovered (Figure 2H).

#### Case 2

**Figure 3:**
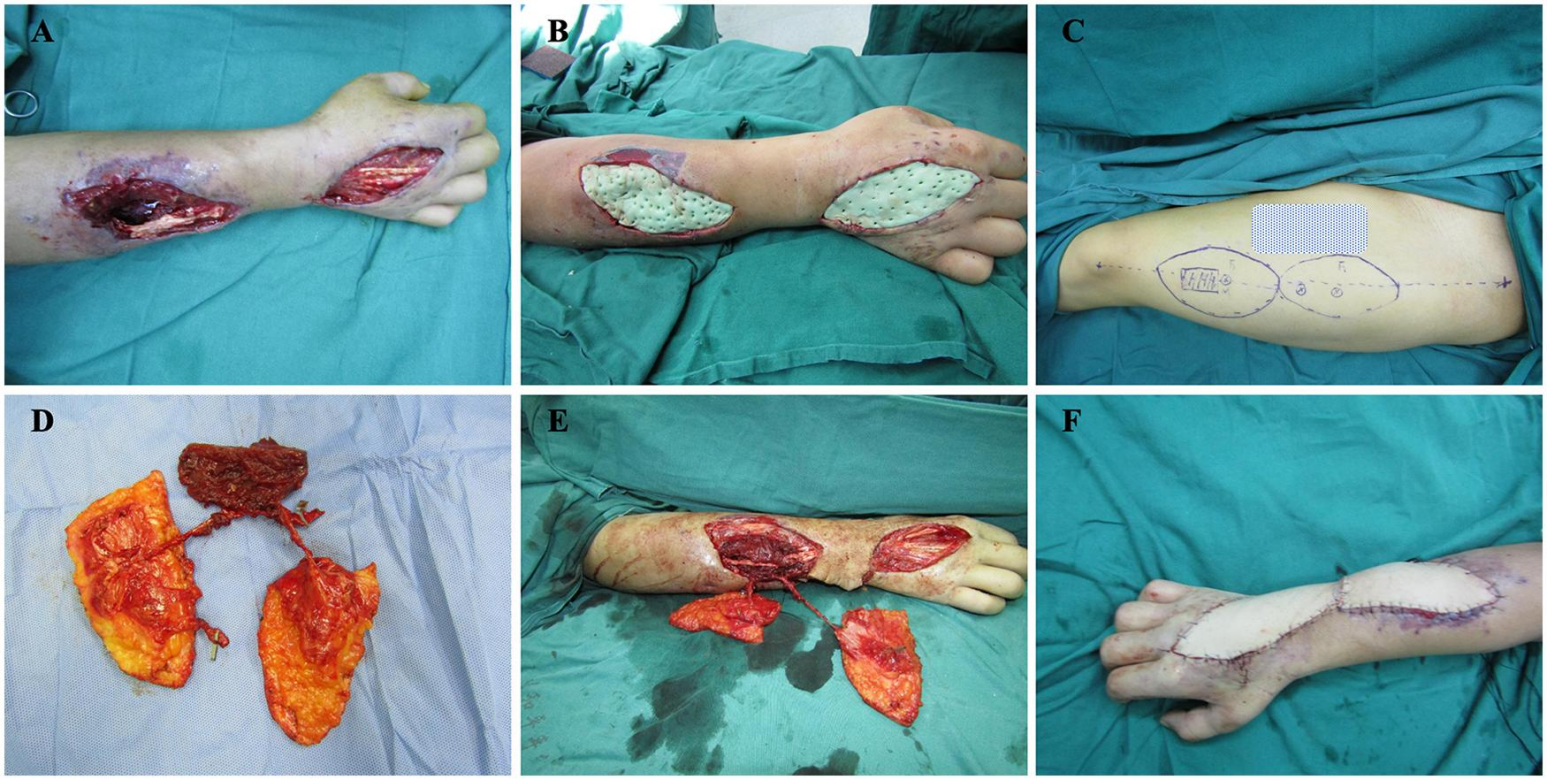
Combined application of the ALT-CMF for reconstruction of forearm and hand CTD. **(A)** Traumatic injury resulting in skin and partial muscle defects of the forearm, accompanied by dorsal hand soft-tissue loss with exposed tendons; **(B)** Placement of antibiotic-loaded bone cement for temporary coverage and infection control; **(C,D)** Preoperative design and intraoperative harvest of the ALT-CMF; **(E,F)** Immediate postoperative appearance of the right forearm after ALT-CMF transfer; skin between the wound areas was excised to avoid compression of the vascular pedicle.

**Figure 4:**
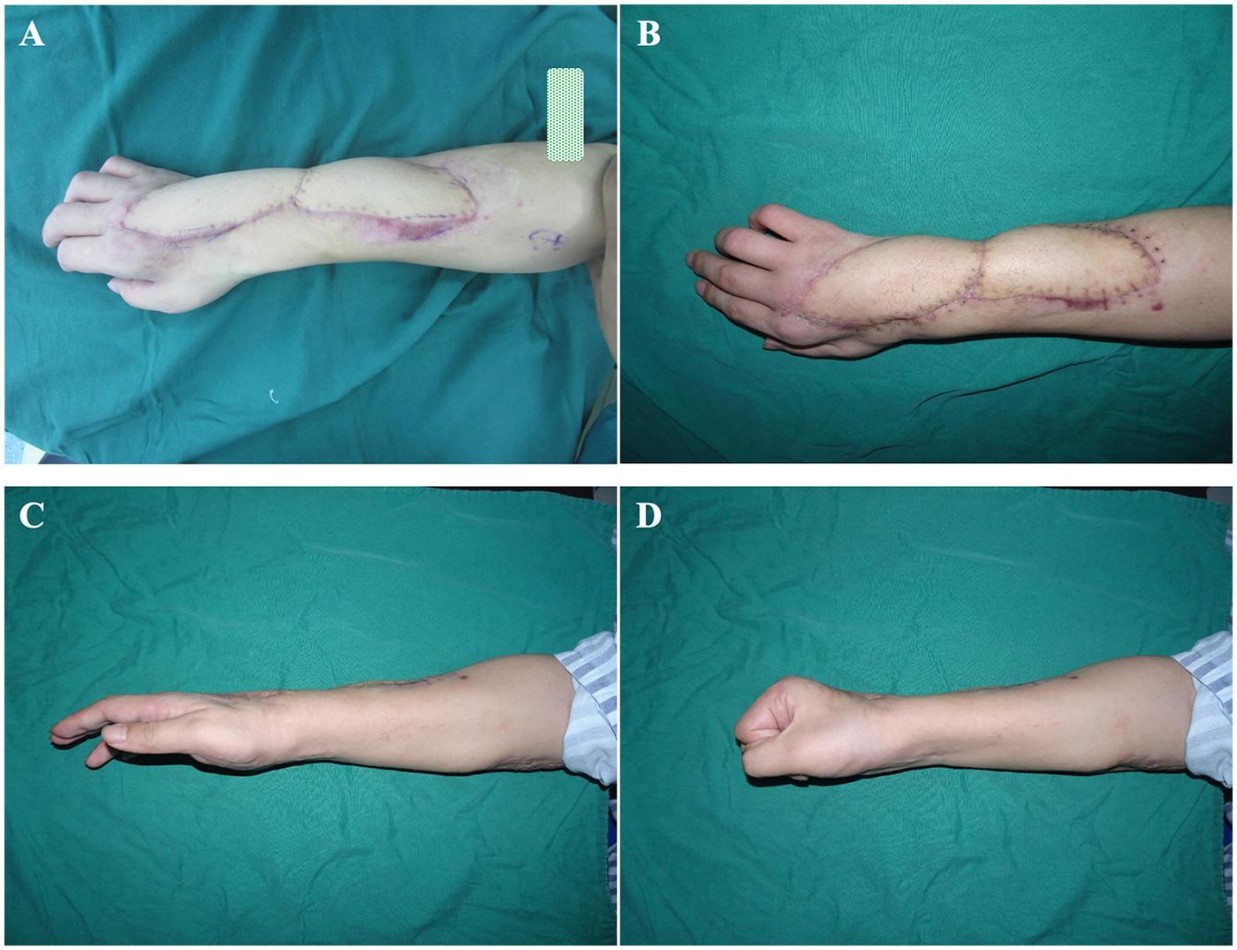
Outcomes of ALT-CMF reconstruction for forearm and hand CTD. **(A)** The flap showed complete survival one month after surgery; **(B)** At one year postoperatively, the transferred flap remained stable without complications; **(C,D)** Satisfactory recovery of basic hand function at one-year follow-up.

A 36-year-old man sustained traumatic composite tissue defects involving the right forearm and dorsal hand (Figure 3A). After thorough debridement, antibiotic-loaded bone cement was applied for temporary coverage and infection control (Figure 3B). Following two weeks of anti-infective treatment, a free anterolateral thigh flap with a vascularized muscle component was harvested from the left thigh (Figures 3C,D). To prevent compression of the vascular pedicle, the skin bridge between the two wound areas was excised, and the chimeric flap components were inset accordingly (Figures 3E,F). One month postoperatively, the flap and surrounding skin showed no signs of necrosis (Figure 4A). At one year of follow-up, both the forearm and hand exhibited satisfactory appearance and functional recovery, with the patient able to perform routine hand and finger movements (Figures 4B–D).

## Discussion

The management of CTD of the forearm and hand represents a complex clinical challenge. These injuries, often caused by high-energy trauma, involve multiple structures—including skin, muscle, nerves, and vessels—with muscle loss posing a particularly significant threat to limb function. Such defects are not merely superficial soft-tissue losses; rather, they require restoration of functional muscular support and reconstruction of neurovascular structures. Therefore, precise and comprehensive reconstructive strategies are essential.

The flexor and extensor muscle groups of the forearm, together with the intrinsic thenar and hypothenar muscles, are critical for wrist and hand motion. Injury to these muscle units leads to substantial functional impairment. Traditional reconstructive approaches relying on single tissue transfer often fail to address the multidimensional structural and functional requirements of these complex defects.

The concept of the chimeric perforator flap was first proposed by Hallock, who pioneered the clinical application of the chimeric flap based on the lateral circumflex femoral artery system (14). As a multi-component reconstructive option, the chimeric perforator flap offers robust vascularity and strong resistance to infection. With only a single vascular pedicle to anastomose, it enables true three-dimensional reconstruction of complex wounds. Owing to its structural versatility and flexible design, it has emerged as a valuable technique in the repair of composite defects (22).

However, reports on the use of the ALT-CMF for reconstruction of forearm and hand CTD remain limited. In our series, this technique enabled successful restoration of the injured muscle groups in the forearm and hand. Postoperatively, patients regained the ability to perform multiple functional tasks, such as pushing and grasping, with overall improvement in hand function. Overall limb contour, functional performance, and sensory recovery were satisfactory.

It should be noted that associated injuries, including nerve damage and bone defects, were unevenly distributed among patients in this cohort. These factors are well recognized to have a substantial impact on functional recovery of the forearm and hand. However, due to the limited sample size and marked heterogeneity in injury type and severity, a subgroup analysis to evaluate the independent effects of nerve injury or bone loss on postoperative functional outcomes was not feasible in the present study.

The ALT-CMF offers several important advantages in the reconstruction of CTD of the forearm and hand. This technique requires dissection and anastomosis of only a single vascular pedicle, thereby minimizing donor-site morbidity and reducing operative time. It is technically straightforward, as the cutaneous perforators from the descending branch of the lateral circumflex femoral artery also supply the vastus lateralis, allowing partial harvest of the muscle without compromising thigh function. The muscle component can be precisely tailored to match the extent of muscle loss, enabling effective obliteration of dead space while providing functional muscular support to the affected limb.

Moreover, the versatility of this approach permits flexible incorporation of fascia, muscle, and nerve components based on defect characteristics, allowing simultaneous soft-tissue coverage, restoration of motor function, and sensory reconstruction. Although the inter-component pedicle length was not routinely measured, based on intraoperative experience it generally ranged from approximately 3–8 cm. This length varied according to the size and location of the recipient defect, as well as the intramuscular course of the vascular pedicle, and was sufficient to allow flexible three-dimensional arrangement of the skin and muscle components.Together, these features enable a true one-stage, comprehensive repair for complex forearm and hand defects.

In the present study, the decision to incorporate a muscle component was based on intraoperative assessment of dead space rather than on a predefined volumetric threshold (23, 24). Dead space was considered clinically significant when extensive three-dimensional cavities remained after thorough debridement, particularly in the presence of exposed bone, tendons, or hardware, or when soft-tissue collapse was anticipated to compromise wound stability (25). In such cases, a muscle component was incorporated to provide bulk for dead space obliteration and to enhance wound stability. Conversely, when the defect was superficial with minimal dead space and adequate coverage could be achieved using a fasciocutaneous component alone, incorporation of a muscle component was deemed unnecessary.

Although the surface defect in the thenar region shown in some cases appeared relatively limited, intraoperative exploration revealed deeper tissue loss and potential dead space that could not be adequately addressed by a fasciocutaneous flap alone. For example, in case 1, incorporation of a muscle component was considered necessary to provide volumetric support, enhance wound stability, and prevent postoperative soft tissue collapse. Simpler reconstructive options were carefully considered; however, they were deemed insufficient to simultaneously address the deep dead space and functional requirements of the thenar region. Therefore, a chimeric flap design was selected to achieve a more reliable and durable reconstruction.

It is worth noting that preservation and reconstruction of muscle function are critical intraoperative steps, as they directly influence postoperative limb function and overall quality of life. Proper tailoring and secure fixation of the muscle flap are essential to maintaining physiological muscle tension and ensuring functional recovery. By accurately assessing the extent of flexor muscle loss and the condition of the remaining musculature, appropriate shaping and fixation of the muscle flap help facilitate active limb motion after reconstruction.

Rehabilitation plays a pivotal role in the management of CTD of the forearm and hand, with early functional training being particularly crucial for preventing muscle atrophy and restoring flexor function (2, 26, 27). Early rehabilitation aims to enhance muscle perfusion and promote neurofunctional recovery through passive and active motion, thereby preventing joint contracture and tendon adhesion and laying the foundation for subsequent functional improvement (26, 28, 29). Specific strategies include passive joint mobilization, isometric muscle training, and progressively advanced active exercises. In addition, electrical stimulation targeting the transferred muscle has been employed to facilitate neuromuscular reinnervation and improve functional outcomes.

In chimeric perforator flaps, the muscle component is often located in a position that prevents direct assessment of viability through surface indicators such as color or temperature. The vascular supply of the muscle flap depends largely on the continuous vascular network within the epimysium; however, the epimysium is prone to separation from the underlying muscle fibers (30, 31). Therefore, when harvesting the muscle flap, it is essential to preserve an adequate portion of the epimysium and avoid stripping it from the muscle tissue to ensure sufficient perfusion and flap viability.

Postoperative complications of the ALT-CMF primarily include flap necrosis, infection, and functional impairment of the affected limb. Flap necrosis is one of the most frequently encountered complications, with vascular crisis being a major contributing factor. Such crises typically arise from insufficient arterial inflow, impaired venous outflow, or vasospasm at the anastomotic site. Failure to recognize and manage these events promptly can significantly compromise flap survival and adversely affect the overall reconstructive outcome (32, 33).

The relatively high incidence of vascular crisis observed in our study warrants a cautious root cause analysis. One potential contributing factor lies in the inherent structural complexity of the chimeric flap design. The ALT-CMF requires a relatively long vascular pedicle to simultaneously supply both the skin and muscle components; within the confined anatomical space of the forearm and hand, such a pedicle is more susceptible to kinking or torsion, thereby increasing the risk of compromised blood flow (34). In addition, in cases complicated by arterial defects, the use of flow-through anastomosis may require the reconstructed vessel to simultaneously provide distal limb perfusion and flap blood supply, potentially imposing an increased early hemodynamic burden (35). Taken together, although the ALT-CMF offers considerable flexibility in the reconstruction of composite tissue defects, its structural configuration and hemodynamic characteristics may represent potential limitations of this technique, thereby increasing the risk of vascular complications.

In our study, five cases of vascular crisis were identified, and one flap ultimately progressed to complete necrosis despite salvage attempts. Close postoperative monitoring of flap perfusion is essential for preventing vascular compromise. Common monitoring modalities include handheld Doppler assessment of arterial signals, laser Doppler flowmetry, near-infrared spectroscopy to evaluate microcirculatory perfusion, and clinical evaluation of flap color, temperature, and capillary refill. Warning signs such as flap darkening, reduced temperature, or diminished Doppler signals should raise strong suspicion of impending vascular crisis. Prompt bedside interventions—such as releasing tension, evacuating hematoma, or optimizing circulation—and timely re-exploration when necessary, can markedly improve flap salvage rates.

Published evidence indicates that selecting an appropriate anastomotic strategy and employing meticulous microsurgical technique significantly reduces the incidence of flap necrosis, while strict postoperative surveillance facilitates early detection of perfusion impairment. Moreover, timely salvage measures can prevent flap failure and improve overall reconstructive outcomes (36–38).

Infection represents another major postoperative complication in these complex injuries, particularly in cases with extensive wounds, heavy contamination, or exposed bone or tendons. Infection can lead to tissue necrosis, functional impairment, and, in some instances, the need for secondary surgical intervention (39–41). Effective preventive strategies include strict adherence to aseptic technique, thorough intraoperative debridement and tissue management, appropriate postoperative antibiotic administration, and early recognition of infection-related signs. For patients who develop postoperative infection, timely surgical debridement combined with targeted antimicrobial therapy is essential to control infection, preserve the viability of the transferred tissues, and support functional recovery. High-risk patients—such as those with diabetes—require intensified postoperative monitoring and management to further reduce the risk of infectious complications (20, 42).

Functional impairment is another important postoperative concern, typically presenting as limited digital flexion and extension, tendon adhesion, and muscle atrophy. Following flexor tendon reconstruction, adhesion formation is a major cause of functional limitation, as it restricts tendon gliding and reduces joint range of motion (43, 44). Early postoperative rehabilitation is therefore essential. Evidence suggests that initiating controlled active motion within 3–5 days after tendon repair can effectively reduce adhesion formation and joint stiffness, thereby enhancing functional recovery (26, 45). In addition, the techniques used for muscle and tendon repair have a direct impact on postoperative outcomes; selecting appropriate repair methods and implementing optimal postoperative management strategies can further reduce the incidence of functional deficits.

It should be emphasized that the improvement in digital motion observed in this study was primarily attributable to adequate tenolysis, appropriate tendon repair, and the establishment of a stable wound bed, which together facilitated effective tendon gliding and postoperative rehabilitation, rather than to any active pulling force generated by the transferred muscle itself.

## Limitations

This study has several limitations. First, this was a single-center retrospective study with a relatively small sample size and no control group, which may limit the generalizability of the findings. Second, substantial heterogeneity existed among patients with respect to injury mechanisms, extent of tissue loss, associated nerve or bone injuries, and preoperative management, including differences in wound contamination and reconstruction timing. These factors may have introduced confounding effects that could not be fully controlled.

Third, although associated nerve injuries and bone defects were documented, the limited sample size and uneven distribution precluded meaningful subgroup analyses to assess their independent impact on functional outcomes. Similarly, the study was not powered to evaluate the effect of reconstruction timing on clinical results. Finally, postoperative functional assessment primarily relied on Total Active Motion (TAM), which mainly reflects joint mobility and does not fully capture other important domains of functional recovery, such as muscle strength, dexterity, and patient-reported outcomes. Therefore, functional conclusions should be interpreted with appropriate caution.

## Conclusion

This study summarizes our clinical experience using the free anterolateral thigh chimeric muscle flap for the reconstruction of CTD of the forearm and hand. The findings demonstrate that this technique provides reliable soft-tissue coverage, achieves favorable flap survival and wound-healing outcomes, and is particularly well suited for upper-extremity injuries involving complex anatomy and multilayered tissue loss. Moreover, satisfactory functional outcomes in terms of joint mobility and sensation were achieved, supporting the broader clinical application of the ALT-CMF and lay the groundwork for future large-scale, multicenter studies to further validate its efficacy.

## Data Availability

The original contributions presented in the study are included in the article/Supplementary Material, further inquiries can be directed to the corresponding authors.
